# Cadmium activation of wild-type and constitutively active estrogen receptor alpha

**DOI:** 10.3389/fendo.2024.1380047

**Published:** 2024-08-09

**Authors:** John B. Psaltis, Qiaochu Wang, Gai Yan, Reem Gahtani, Nanxi Huang, Bassem R. Haddad, Mary Beth Martin

**Affiliations:** ^1^ Department of Oncology, Georgetown University, Washington, DC, United States; ^2^ Depatment of Biochemistry and Molecular & Cellular Biology, Georgetown University, Washington, DC, United States

**Keywords:** estrogen receptor, metals, metalloids, cadmium, constitutively active mutants, Y537S, D538G

## Abstract

The estrogen receptor alpha (ERα) plays a central role in the etiology, progression, and treatment of breast cancers. Constitutively activating somatic mutations Y537S and D538G, in the ligand binding domain (LBD) of *ESR1*, are associated with acquired resistance to endocrine therapies. We have previously shown that the metalloestrogen calcium activates ERα through an interaction with the LBD of the receptor. This study shows that cadmium activates ERα through a mechanism similar to calcium and contributes to, and further increases, the constitutive activity of the ERα mutants Y537S and D538G. Mutational analysis identified C381, N532A, H516A/N519A/E523A, and E542/D545A on the solvent accessible surface of the LBD as possible calcium/metal interaction sites. In contrast to estradiol, which did not increase the activity of the Y537S and D538G mutants, cadmium increased the activity of the constitutive mutants. Mutation of the calcium/metal interaction sites in Y537S and D538G mutants resulted in a significant decrease in constitutive activity and cadmium induced activity. Mutation of calcium/metal interaction sites in wtERα diminished binding of the receptor to the enhancer of estrogen responsive genes and the binding of nuclear receptor coactivator 1 and RNA polymerase II. In contrast to wtERα, mutation of the calcium/metal interaction sites in the Y537S and D538G mutants did not diminish binding to DNA but prevented a stable interaction with the coactivator and polymerase. Growth assays further revealed that calcium channel blockers and chelators significantly decreased the growth of MCF7 cells expressing these constitutively active mutants. Taken together, the results suggest that exposure to cadmium plays a role in the etiology, progression, and response to treatment of breast cancer due, in part, to its ability to activate ERα.

## Introduction

Breast cancer is the most commonly diagnosed and second leading cause of cancer-associated death in women (1). Approximately 70% of breast tumors are estrogen receptor (ER) positive at the time of diagnoses, but approximately 30% fail endocrine therapy (2). Environmental exposures to endocrine disruptors that mimic the biological effects of estrogens are linked to the high incidence of breast cancer but may also contribute to progression and treatment failure. Because the estrogen receptor plays a central role in breast cancer, understanding the mechanisms by which ERα is activated is critical to designing more effective prevention and treatment strategies.

Metalloestrogens are metals and metalloids that activate ERα independent of estradiol and include the bivalent cations cadmium and calcium. Our previous studies show that the second messenger calcium mediates the activation of ERα by epidermal growth factor through four sites on the solvent accessible surface of the ligand binding domain (LBD) (3). Our previous studies also show that cadmium, which mimics calcium, activates ERα independent of estradiol (4, 5) and promotes the development of mammary tumors (6). Cadmium is also linked to an increased risk of developing breast cancer (7–9). The ability of calcium to activate ERα provides a possible explanation for the ability of metals to activate the receptor (4, 10, 11) suggesting that metals mimic a physiological activator of ERα to increase the risk for developing the disease (12, 13).

Several mechanisms are thought to be responsible for endocrine resistant breast cancer including hormone independent activation of ERα by growth factor signaling pathways (14, 15) and mutation or loss of the receptor (16). Approximately 25-30% of endocrine resistance is linked to mutations in the LBD of ERα (17–20). The most prevalent mutations are Y537S and D538G that confer hormone independence and resistance by favoring an agonist like conformation of the LBD that results in reduced binding of ligands and efficacy of tamoxifen, a selective estrogen receptor modulator, and fulvestrant, a selective estrogen receptor degrader (17–22).

Although there is evidence linking cadmium to the risk of developing breast cancer, the mechanism by which cadmium activates ERα and contributes to endocrine resistance is not fully understood. The goal of the present study is to gain insight into the mechanism by which cadmium activates wild-type ERα (wtERα) and the constitutively active ERα mutants Y537S and D538G. This study asks whether cadmium activates ERα through a mechanism similar to calcium (3) and whether the calcium/metal interaction sites contribute to the constitutive activity of Y537S and D538G. The results presented herein demonstrate that the sites necessary for calcium activation of ERα are also necessary for cadmium activation of the receptor. In addition, mutation of calcium/metal interaction sites in ERα mutants Y537S and D538G leads to a significant reduction in constitutive activity and activation by cadmium. Mutation of calcium/metal interaction sites in wtERα diminished binding to DNA and binding of SRC1 and PolII whereas mutation of the calcium/metal binding site in the constitutively active mutants did not completely diminish binding to DNA but abrogated the recruitment of coregulators. Together, the results suggest that the constitutive activity of ERα mutants Y537S and D538G is due, in part, to the interaction of cadmium with calcium/metal interaction sites on ERα. As the metal interaction sites in this study were previously shown to be important for calcium (3), the results also suggest that the constitutive activity of Y537S and D538G may be mediated, in part, by calcium.

## Materials and methods

### Reagents

Cadmium Chloride (cat#C202908) and 17β-estradiol (cat#E8875) were purchased from Sigma-Aldrich, Burlington, MA. ICI-182,780 (cat#10-471) was purchased from Tocris Bioscience, Bristol, UK.

EDTA (cat#V4231) was purchased from Promega. Methoxyverapamil hydrochloride (MV) (cat#M5644) was purchased from Sigma. Mibefradil dihydrochloride (MF) (cat#2198), BAPTA (cat#2786) and BAPTA-AM (cat#2787) was purchased from Tocris. EDTA-AM (cat# 19010) was purchased from AATBIO.

### Cell culture and transient transfection assay

Fingerprinted and authenticated HEK293T cells (RRID: CVCL_0063) were acquired from Dr. Anna Riegel’s laboratory and maintained in lipoic acid-free improved minimal essential medium (IMEM; Crystalgen, Commack, NY) supplemented with 10% fetal bovine serum (FBS; Sigma-Aldrich). For transfection assays, 5×10^5^ cells were plated in six-well plates in phenol red- and lipoic acid-free IMEM containing 5% charcoal stripped serum (CSS; Sigma-Aldrich). Twenty-four hours later, cells were transfected using *Trans*-IT LT-1 (MirusBio, Madison, WI) at a ratio of 3µl to 1µg of DNA. A total of 5 µg of plasmid DNA was added. After 24 hours, the medium was diluted to 1% CSS for 48 hours prior to treatment.

### Cell culture and MCF7 Y537S and MCF7 D538G growth assays

Fingerprinted and authenticated MCF7 Y537S and MCF7 D538G were gifts from Dr. Joyce Slingerland’s lab, Georgetown University (23). The cells were carried in DMEM with 10% FBS. To start, 1x10^4^ cells were split into 96 wells in 200 µl IMEM with 5% CSS. 24 hours later, serum was diluted to 1% CSS and cells were treated.

MCF7 Y537S and MCF7 D538G cells were washed with PBS. Then 50 µl crystal violet was added to each well and cells were incubated for 20 minutes at room temperature on a rocker. Plates were then washed with tap water three times and air dried overnight. 200 µl methanol was added and cells were incubated for 20 minutes at room temperature on a rocker. A plate reader was used to read the cell confluence.

### Real time polymerase chain reaction

RNA was isolated using Trizol (Life Technologies, Carlsbad, CA). The reverse transcription reaction was performed using Thermo Scientific Maxima™ H Minus cDNA Synthesis Master Mix with dsDNase (Thermo Fisher Scientific, Waltham, MA). qPCR assays were performed using Taqman probe (Thermo Fisher Scientific), SsoAdvanced Universal Probes supermix (Bio-Rad Laboratories, Hercules, CA) or PowerUp™ SYBR™ Green Master Mix (Thermo Fisher Scientific). qPCR results were normalized to ribosomal protein P0 (RPLP0) mRNA and presented as fold change using the 2^-ΔΔct method.

### Site-directed mutagenesis

QuikChange Lightning Site-Directed Mutagenesis Kit (Agilent, Santa Clara, CA) was used. Mutation primers were designed using Agilent QuikChange Primer Design Tool and synthesized by Integrated DNA Technologies. Mutants of pCMV-hERalpha (RRID: Addgene_101141) and previous ERα mutants were verified by sequencing. ERα mutants C381A, H516A, E523A, N532A, D538A, and E542A/D545A are described elsewhere (3, 24).

Y537S mutation primers:

Forward: 5’-ACGTGGTGCCCCTCAGTGACCTGCTGCTGG-3’

Reverse: 5’-CCAGCAGCAGGTCACTGAGGGGCACCACGT -3’

D538G mutation primers:

Forward: 5’-GTGCCCCTCTATGGCCTGCTGCTGGCG-3’

Reverse: 5’-CGCCAGCAGCAGGCCATAGAGGGGCAC-3’

N519A mutation primers:

Forward: 5’-CCACATCAGGCACATGAGTGCCAAAGGCATGGAGCATCTG-3’

Reverse: 5’-CAGATGCTCCATGCCTTTGGCACTCATGTGCCTGATGTGG-3’

E523A mutation primers:

Forward: 5’-GAGTAACAAAGGCATGGCGCATCTGTACAGCATGA-3’

Reverse: 5’-TCATGCTGTACAGATGCGCCATGCCTTTGTTACTC-3’

### Chromatin immunoprecipitation assays

Cells were treated with 100nM estradiol or 2µM cadmium for 1 hour, crosslinked with 1% formaldehyde for 5 minutes, washed twice with PBS, collected in PBS containing protease and phosphatase inhibitors, lysed with nuclei isolation buffer (50mM TrisCl, 60mM KCL, 0.5%NP40, 10mM DTT) containing protease and phosphatase inhibitors, and centrifuged. Nuclei were lysed with lysis buffer (#20-163 EMD Millipore, Burlington, MA) containing protease and phosphatase inhibitors. The chromatin was sonicated, diluted with dilution buffer (#20-153 EMD Millipore; 1:9), and precleared with protein A/G magnetic beads (Pierce, Waltham, MA Cat#88802). ERα was immunoprecipitated with anti-ERα antibody (Santa Cruz Biotechnology, Dallas, TX Cat#sc-8002, RRID: AB_627558) or IgG (Abcam, Cambridge, UK Cat#ab18413, RRID: AB_2631983) overnight and incubated with precleared protein A/G magnetic beads. The beads were washed once with low salt buffer, twice with high salt buffer, once with LiCl buffer (#20-154, -155, & -156 EMD Millipore), once with TE buffer, and eluted with buffer (1%SDS, 100mM NaHCO_3_). To reverse crosslink, 5M NaCl was added, incubated at 65°C overnight, and treated with RNaseA (#46-7604 Invitrogen, Waltham, MA). DNA was purified using the QIAquick PCR purification kit (#28104 Qiagen, Hilden, Germany) and quantified by real time qPCR using PowerUp SYBR Green Master Mix (Thermo Fisher Scientific). For re-ChIP, the second immunoprecipitation was performed with antibodies to SRC1 (Abcam Cat#ab2859, RRID: AB_303360) or PolII (Thermo Fisher Scientific Cat#MA1-26249, RRID: AB_795353).

Promoter primers [Invitrogen (25)]:

hC3-fwd 5’-GAGAAAGGTCTGTGTTCACCAGG-3’,

hC3-rev 5’-TGCAGGGTCAGAGGGACAGA-3’,

LTF-fwd 5’-AGAGTCAAGACCAGCTTTTCAGA-3’, and

LTF-rev 5’-AACACCTTTCTTGGCAGGTGAG-3’.

### Western blot

Forty-eight hours post-transfection, HEK293 cells were washed with PBS and lysed with radioimmunoprecipitation assay buffer (500mM NaCl, 1% NP-40, 0.5% sodium deoxycholate, 0.1% SDS, 50mM Tris, pH 8.0, 2mM leupeptin; Roche, Basel, Switzerland) and incubated on ice for 15 minutes. Protein concentration was determined using Bio-Rad Protein Assay Dye Reagent (Bio-Rad Laboratories). After centrifuge, cell lysates were run on a 10% SDS-PAGE gel, transferred to nitrocellulose, and incubated with anti-ERα antibody or anti-actin antibody (EMD Millipore Cat#MAB1501, RRID: AB_2223041; 1:2000) followed by anti-mouse IgG (SeraCare Life Sciences, Milford, MA Cat#5220-0341, RRID: AB_2891080; 1:10000). Proteins were visualized on Amersham Imager 600 (GE Healthcare) using Western Lightening^®^ Chemiluminescence Reagent Plus (Perkin-Elmer, Waltham, MA).

### Gene set enrichment analysis

Raw microarray data (accession no. GSE136595) was downloaded from Gene Expression Omnibus (GEO, RRID: SCR_005012). Analysis of microarray data was performed by GSEA (RRID: SCR_003199) v4.1.0. Hallmark gene sets were used to determine the molecular signature.

### Statistical analysis

Statistical analyses were performed in Prism. Data are presented as the mean ± standard error of the mean (SEM). Statistical differences were evaluated by one-way ANOVA followed by Fisher’s LSD test. Statistical significance is defined as a P value of ≤ 0.05. < ≤ 0.05*, < ≤ 0.01**, <0.001***. ≤

## Results

### Effect of cadmium on early and late estrogen response in MCF-7 cells

**Figure 1:**
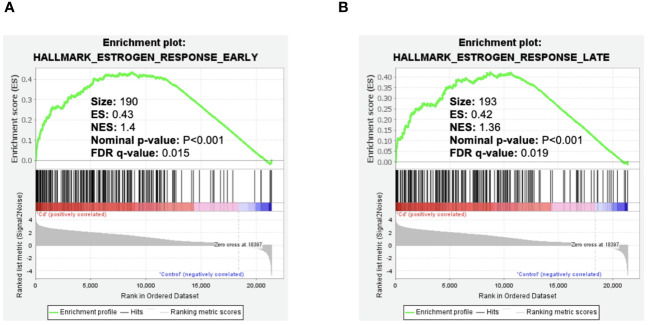
Effect of cadmium on early and late estrogen response in MCF-7 cells. Gene enrichment plots were determined by GSEA of GSE136595. **(A)** Cadmium effect on early estrogen response genes. **(B)** Cadmium effect on late estrogen response genes.

To ask whether cadmium influences early and late estrogen response pathways, data from our published study (6) in MCF7 cells treated with cadmium were reanalyzed. GSEA analysis indicated significant increases of Hallmark Estrogen Response Early/Late pathways (**Figure 1**) which is consistent with our previously published studies showing that cadmium activates ERα (4).

### Effects of cadmium on estrogen responsive genes in HEK293T cells transfected with wild-type and LBD mutants of ERα

**Figure 2:**
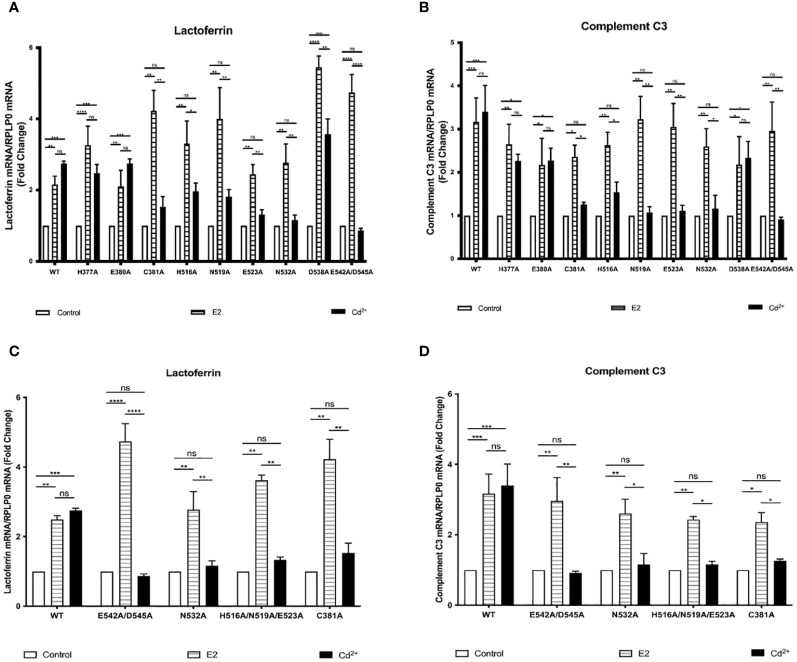
Effects of cadmium on estrogen responsive genes in HEK293T cells transfected with wild-type and LBD mutants of ERα. HEK293T cells were transiently transfected with wtERα, H377A, E380A, C381A, H516A, N519A, E523A, N532A, D538A, E542A/D545, and calcium/metal interaction site mutants C381A, H516A/N519A/E523A, N532A, and E542A/D545A. Following transfection, the cells were treated with estradiol (100 nM) or cadmium (2 µM) for 48 hours. The RNA was isolated, the amount of lactoferrin and complement C3 mRNA was measured by real time qPCR and normalized to the amount of ribosomal protein P0 (RPLP0) mRNA. Using the 2^^-ΔΔCt^ method, data are presented as fold change compared to control, mean ± SEM; n=3. ≤ ≤ ≤ ≤ Statistical significance is defined as a P value of ≤ 0.05. <0.05*, <0.01**, <0.001***, 0.0001****. **(A)** Effect of cadmium on wtERα, H377A, E380A, C381A, H516A, N519A, E523A, N532A, D538A, and E542A/D545 on lactoferrin. **(B)** Effect of cadmium on wtERα, H377A, E380A, C381A, H516A, N519A, E523A, N532A, D538A, and E542A/D545 on complement C3. **(C)** Effect of cadmium on wtERα and calcium/metal interaction site mutants C381A, H516A/N519A/E523A, N532A, and E542A/D545 on lactoferrin. **(D)** Effect of cadmium on wtERα and calcium/metal interaction site mutants C381A, H516A/N519A/E523A, N532A, and E542A/D545 on complement C3.

Our previous mutational analysis shows that calcium activates ERα through several amino acids on the aqueous surface of the LBD. To determine whether cadmium activates ERα through a similar mechanism, HEK293T cells were transfected with wtERα or ERα LBD mutants H377A, E380A, C381A, H516A, N519A, E523A, N532A, D538A, or E542A/D545A. Following transfection, the cells were treated with estradiol or cadmium for 24 hours and the amount of the ERα target genes lactoferrin and complement C3 mRNA was measured (**Figure 2**). Wild-type ERα was treated with cadmium and the antiestrogen ICI-182,780 (fulvestrant; 1µM) (**Supplementary 3**). As expected, estradiol treatment of cells transfected with wild-type and ERα LBD mutants resulted in an approximately 2.15- to 5.45-fold increase in lactoferrin and complement C3 mRNA. In cells transfected with wtERα, cadmium treatment resulted in an approximately 2.75- and 3.40-fold increase in lactoferrin and complement C3 mRNA, respectively, that was blocked by ICI-182,780. In cells transfected with H377A, E380A, and D538A, treatment with cadmium also resulted in an approximately 2.26- to 3.57-fold increase in lactoferrin and complement C3 mRNA. However, in cells transfected with the C381A, H516A, N519A, E523A, N532A, and E542A/D545A mutants, treatment with cadmium failed to increase the expression of lactoferrin and complement C3 suggesting that amino acids C381, H516, N519, E523, N532, and E542/D545 are important for cadmium activation of ERα.

### Effects of cadmium on ERα wild-type and calcium/metal interaction site mutants in HEK293T cells

Our previous mutational analysis combined with binding assays and molecular modeling (3) identified four potential calcium interaction sites on ERα that involve amino acid side chain and backbone interactions. To determine whether cadmium activates ERα through calcium interaction sites, mutants E542A/D545A, H516A/N519A/E523A, N532A, and C381A were constructed. HEK293T cells were then transfected with wtERα or the calcium interaction site mutants, treated with estradiol or cadmium, and the amount of lactoferrin and complement C3 mRNA was measured (**Figures 2C, D**). As expected, estradiol treatment of the wild-type ERa or ERα calcium interaction site mutants resulted in a 2.49- to 4.73-fold increase in lactoferrin and complement C3. Treatment of wtERα with cadmium also resulted in 2.75- and 3.40-fold increase in lactoferrin and complement C3, respectively. However, treatment of the calcium/metal interaction site mutants E542A/D545A, N532A, H516A/N519A/E523A, and C381A with cadmium failed to induce lactoferrin and complement C3 suggesting that the interaction of cadmium at the calcium interaction sites is necessary for cadmium activation of ERα.

To determine if there were differences in expression of wild-type ERa and the calcium/metal interaction site mutant ERα in HEK293T cells, a western blot was performed (**Supplementary 1**). There was no significant difference in expression and, as expected, untransfected cells did not express ERα.

### Effects of cadmium on the recruitment of ERα wild-type and calcium/metal interaction site mutants to the enhancers of estrogen responsive genes

**Figure 3:**
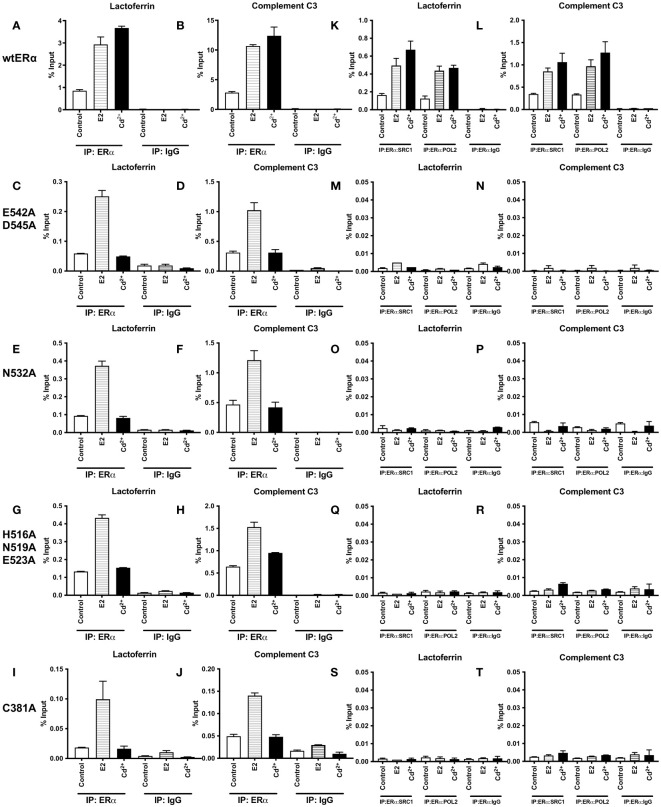
Effects of cadmium on ERα wild-type and calcium/metal interaction site mutants in HEK293T cells. HEK293T cells were transfected with wild-type ERα and mutants E542A/D545A, N532A, H516A/N519A/E523A, and C381A. Following transfection, the cells were treated with estradiol (100 nM) or cadmium (2 µM) for 60 minutes, crosslinked, lysed, and the occupancy of ERα on the enhancers of target genes was examined by ChIP assay. A Re-ChIP assay was performed, and the chromatin was first immunoprecipitated with an antibody to ERα, followed by immunoprecipitation with an antibody to SRC1, PolII, or IgG. DNA was quantified by real time qPCR and the recruitment of ERα, SRC1, and PolII is presented as percent input. The data are the mean of two independent experiments done in duplicate (mean ± SEM). **(A, B)** ChIP wtERα. **(C, D)** ChIP E542A/D545A. **(E, F)** ChIP N532A. **(G, H)** ChIP H516A/N519A/E523A. **(I, J)** ChIP C381A. **(K, L)** Re-ChIP wtERα. **(M, N)** Re-ChIP E542A/D545A. **(O, P)** Re-ChIP N532A. **(Q, R)** Re-ChIP H516A/N519A/E523A. **(S, T)** Re-ChIP C381A.

To determine whether cadmium treatment alters the recruitment of wild-type and calcium/metal interaction site mutants of ERα to the enhancers of estrogen responsive genes, HEK293T cells were transfected with wtERα or ERα mutants E542A/D545A, N532A, H516A/N519A/E523A, and C381A and treated for 60 minutes with estradiol or cadmium. ERα-DNA complexes were isolated and the recruitment of ERα to the enhancer regions of lactoferrin and complement C3 was quantified by real time qPCR (**Figures 3A–J**). Treatment of wtERα with estradiol resulted in an approximately 3.44- and 3.79-fold increase in enrichment of ERα on the enhancers of lactoferrin and complement C3, respectively. Treatment of the ERα calcium/metal interaction site mutants E542A/D545A, N532A, H516A/N519A/E523A, and C381A with estradiol also resulted in an approximately 2.38- to 5.52-fold increase in enrichment of the mutants on the enhancers of lactoferrin and complement C3. Similar to treatment with estradiol, treatment of wtERα with cadmium resulted in an approximately 4.31- and 4.40-fold increase in enrichment on the enhancers of lactoferrin and complement C3, respectively. However, treatment of ERa calcium/metal interaction site mutants E542A/D545, N532A, H516A/N519A/E523A, and C381A with cadmium did not result in enrichment. As expected, in untransfected cells, there was no enrichment of ERα on the enhancers of lactoferrin or complement C3 in response to estradiol or cadmium (**Supplementary 4**).

To determine whether a transcription complex is formed, a sequential ChIP assay was performed to determine the enrichment of nuclear receptor coactivator 1 (SRC1) and RNA polymerase II (PolII) on the enhancers of lactoferrin and complement C3. Cells were transfected with ERα wild-type or calcium/metal interaction site mutants and treated for 60 minutes with estradiol or cadmium. Immunoprecipitation was performed with the antibody to ERα and then with an antibody to either SRC1 or PolII. The enrichment of SRC1 and PolII was quantified (**Figures 3K–T**). As expected, treatment of wtERα with estradiol resulted in an approximately 3.04- and 2.68-fold increase of SRC1 and PolII on the enhancer of lactoferrin and an approximately 2.47- and 2.90-fold increase of SRC1 and PolII on the enhancer of complement C3, respectively. Similarly, treatment of wtERα with cadmium resulted in approximately 4.12- and 3.78-fold increase of SRC1 and PolII on the enhancer of lactoferrin and an approximately 3.07- and 3.81-fold increase of SRC1 and PolII on the enhancer of complement C3, respectively. However, treatment of ERα mutants E542A/D545, N532A, H516A/N519A/E523A, and C381A with estradiol or cadmium did not result in an increase of SRC1 or PolII on the enhancers of lactoferrin or complement C3. In untransfected cells, there was no enrichment of SRC1 or PolII on the enhancers of lactoferrin or complement C3 in response to estradiol or cadmium (**Supplementary 5**).

### Effects of cadmium on Y537S and Y537S calcium/metal interaction site mutants

To determine whether mutation of ERα calcium/metal interaction sites affects the constitutive activity of Y537S and cadmium induced expression of estrogen responsive genes, the calcium/metal interaction sites in Y537S were mutated. HEK293T cells were transfected with the constitutively active mutant Y537S or the calcium/metal interaction site mutants Y537S/E542A/D545A, Y537S/N532A, Y537S/H516A/N519A/E523A, and Y537S/C381A, treated with estradiol or cadmium, and the amount of lactoferrin and complement C3 was measured. Y537S ERα was additionally treated with cadmium and ICI-182,780.

**Figure 4:**
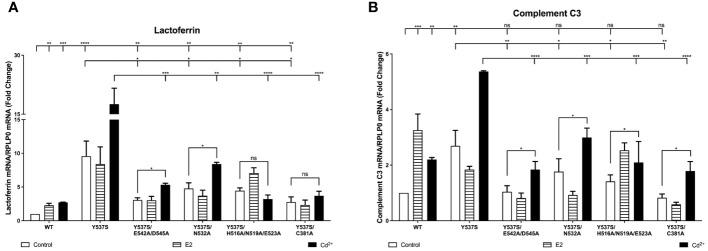
Effects of cadmium on Y537S and Y537S calcium/metal interaction site mutants. HEK293T cells were transiently transfected with wild-type, Y537S, Y537S/E542A/D545A, Y537S/N532A, Y537S/H516A/N519A/E523A, Y537S/C381A. Following transfection, the cells were treated with estradiol (100 nM) or cadmium (2 µM) for 48 hours. The RNA was isolated, the amount of lactoferrin and complement C3 mRNA was measured by real time qPCR and normalized to the amount of ribosomal protein P0 (RPLP0) mRNA. Using the 2^^-ΔΔCt^ method, data are presented as fold change compared to control, mean ± SEM; n=3.≤ ≤ ≤ ≤ Statistical significance is defined as a P value of ≤ 0.05. <0.05*, <0.01**, <0.001***, 0.0001****. **(A)** lactoferrin. **(B)** complement C3.

In the case of expression of lactoferrin, cells transfected with Y537S exhibited a 9.57-fold increase in lactoferrin compared to cells transfected with wtERα, confirming the constitutive activity of Y537S (**Figure 4A**). Compared to treatment of wtERα with cadmium, treatment of Y537S with cadmium resulted in a further 6.46-fold increase in lactoferrin that was blocked by ICI-182,780 (**Supplementary 7A**). Mutation of the calcium/metal interaction sites decreased the constitutive activity of Y537S. Compared to Y537S, there was an approximately 68%, 50%, 54%, and 71% decrease in the constitutive activity of Y537S/E542A/D545A, Y537S/N532A, Y537S/H516A/N519A/E523A, and Y537S/C381A, respectively. Cadmium activation of the calcium/metal interaction site mutants was also significantly lower. There was an approximately 70%, 53%, 82%, and 79% decrease in cadmium activation of Y537S/E542A/D545A, Y537S/N532A, Y537S/H516A/N519A/E523A, and Y537S/C381A, respectively.

In the case of expression of complement C3, cells transfected with Y537S also exhibited an approximately 2.70-fold increase in complement C3 compared to cells transfected with wtERα (**Figure 4B**). Compared to treatment of wtERα with cadmium, treatment of Y537S with cadmium resulted in a further 2.44-fold increase in complement C3 mRNA and the increase was blocked by ICI-182,780 (**Supplementary 7B**). A significant decrease in constitutive activity was also observed upon mutation of the calcium/metal interaction sites. Compared to Y537S, there was an approximately 61%, 45%, 48%, and 70% decrease in the constitutive activity of Y537S/E542A/D545A, Y537S/N532A, Y537S/H516A/N519A/E523A, and Y537S/C381A, respectively. Cadmium activation of the Y537S calcium/metal interaction site mutants was also significantly decreased compared to cadmium activation of Y537S. There was an approximately 66%, 45%, 61%, and 67% decrease in cadmium activation of Y537S/E542A/D545A, Y537S/N532A, Y537S/H516A/N519A/E523A, and Y537S/C381A, respectively.

### Effects of cadmium on the recruitment Y537S and Y537S calcium/metal interaction site mutants to the enhancers of estrogen responsive genes

**Figure 5:**
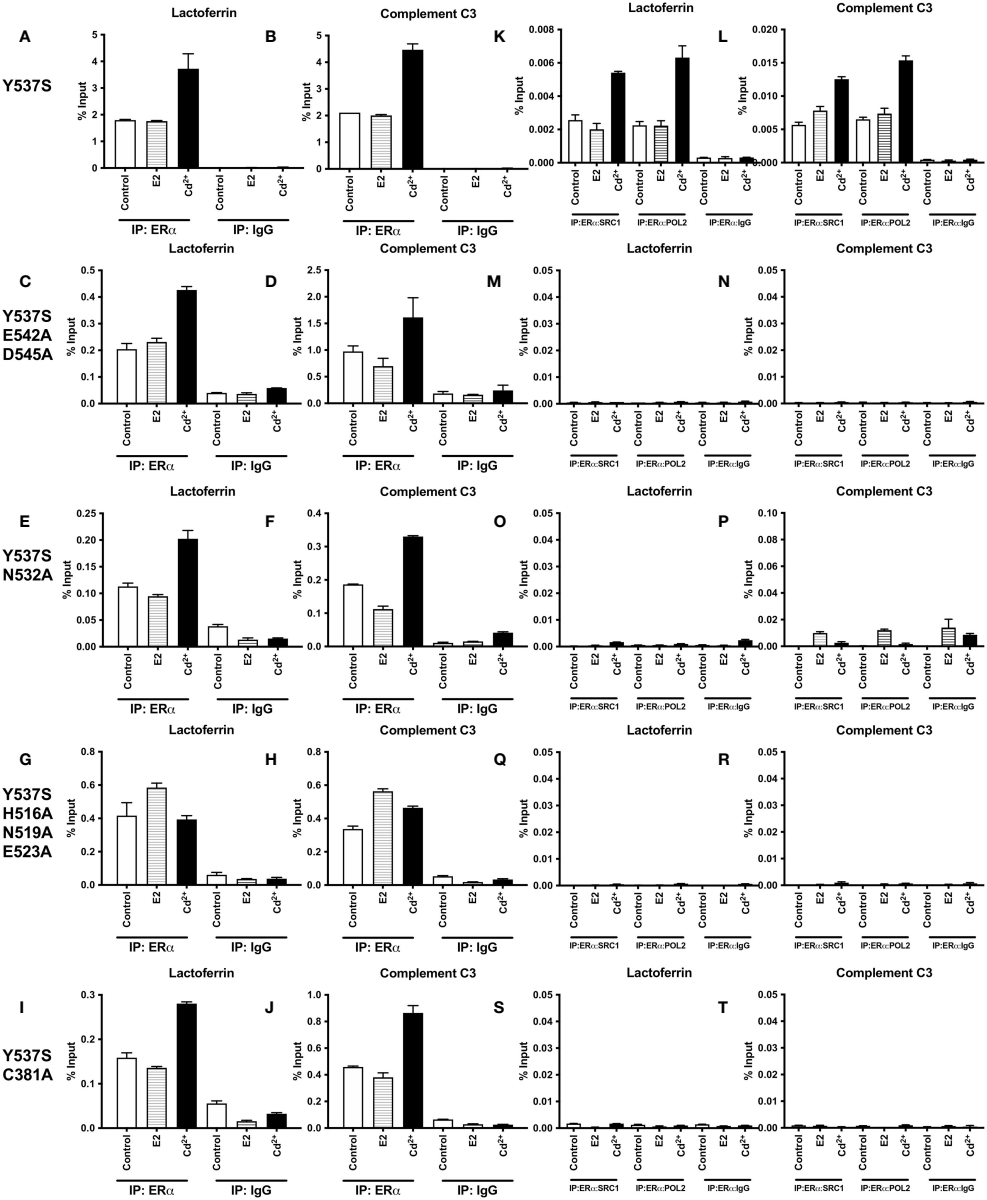
Effects of cadmium on the recruitment of Y537S and Y537S calcium/metal interaction site mutants to the enhancers of estrogen responsive genes. HEK293T cells were transfected with Y537S ERα and mutants Y537S/E542A/D545A, Y537S/N532A, Y537S/H516A/N519A/E523A, and Y537S/C381A. Following transfection, the cells were treated with estradiol (100 nM) or cadmium (2 µM) for 60 minutes, crosslinked, lysed, and the occupancy of ERα on the enhancers of target genes was examined by ChIP assay. A Re-ChIP assay was performed, and the chromatin was first immunoprecipitated with an antibody to ERα, followed by immunoprecipitation with an antibody to SRC1, PolII, or IgG. DNA was quantified by real time qPCR and the recruitment of ERα, SRC1, and PolII is presented as percent input. The data are the mean of two independent experiments done in duplicate (mean ± SEM). **(A, B)** ChIP Y537S. **(C, D)** ChIP Y537S/E542A/D545A. **(E, F)** ChIP Y537S/N532A. **(G, H)** ChIP Y537S/H516A/N519A/E523A. **(I, J)** ChIP Y537S/C381A. **(K, L)** Re-ChIP Y537S. **(M, N)** Re-ChIP Y537S/E542A/D545A. **(O, P)** Re-ChIP Y537S/N532A. **(Q, R)** Re-ChIP Y537S/H516A/N519A/E523A. **(S, T)** Re-ChIP Y537S/C381A.

To determine whether treatment with cadmium alters the recruitment of Y537S and Y537S mutants to the enhancers of estrogen responsive genes, HEK293T cells were transiently transfected with the constitutively active mutant Y537S or the calcium/metal interaction site mutants Y537S/E542A/D545A, Y537S/H516A/N519A/E523A, Y537S/N532A, and Y537S/C381A and treated for 60 minutes with estradiol or cadmium. ERα-DNA complexes were isolated and the recruitment of the mutants to the enhancers of lactoferrin and complement C3 was quantified (**Figures 5A–J**). Compared to control, treatment of Y537S, Y537S/E542A/D545, Y537S/N532A, and Y537S/C381A with estradiol did not increase the enrichment of Y537S or the Y537S calcium/metal interaction site mutants on the enhancers of lactoferrin and complement C3. However, treatment of Y537S with cadmium resulted in an approximately 2.07- and 2.12-fold increase in enrichment of Y537S on the enhancers of lactoferrin and complement C3, respectively. Treatment with cadmium also increased the enrichment of Y537S/E542A/D545, Y537S/N532A, and Y537S/C381A on the enhancers of lactoferrin and complement C3 (~1.70- to 2.09-fold and ~1.77- to 1.89-fold, respectively) but failed to increase the enrichment of Y537S/H516A/N519A/E523A on the enhancers.

To determine whether a transcription complex is formed, a sequential ChIP assay was performed to determine the enrichment of SRC1 and PolII on the enhancers of lactoferrin and complement C3. Cells were transiently transfected with Y537S or the Y537S mutants and treated with estradiol or cadmium. Immunoprecipitation was first performed with the antibody to ERα and then with an antibody to either SRC1 or PolII and the enrichment of SRC1 and PolII was again quantified (**Figures 5K–T**). Treatment of Y537S with estradiol did not further increase the enrichment of SRC1 and PolII on the enhancer of lactoferrin (~0.79- and 0.99-fold, respectively) or complement C3 (~1.37- and 1.13-fold, respectively). In contrast to estradiol, treatment of Y537S with cadmium resulted in approximately 2.11- and 2.80-fold increase in enrichment of SRC1 and PolII on the enhancer of lactoferrin, respectively, and an approximately 2.20- and 2.35-fold increase in enrichment on the enhancer of complement C3, respectively. However, treatment of the calcium/metal interaction site mutants Y537S/E542A/D545, Y537S/N532A, Y537S/H516A/N519A/E523A, and Y537S/C381A with estradiol or cadmium did not increase the enrichment of SRC1 or PolII on the enhancers.

### Effects of cadmium on D538G and D538G calcium/metal interaction site mutants

**Figure 6:**
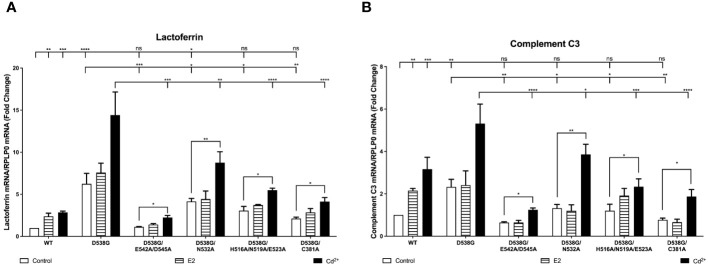
Effects of cadmium on D538G and D538G calcium/metal interaction site mutants. HEK293T cells were transiently transfected with wild-type, D538G, D538G/E542A/D545A, D538G/N532A, D538G/H516A/N519A/E523A, D538G/C381A. Following transfection, the cells were treated with estradiol (100 nM) or cadmium (2 µM) for 48 hours. The RNA was isolated, the amount of lactoferrin and complement C3 mRNA was measured by real time qPCR and normalized to the amount of ribosomal protein P0 (RPLP0) mRNA. Using the 2^^-ΔΔCt^ method, data are presented as fold change compared to control, mean ± SEM; n=3.≤ ≤ ≤ ≤ Statistical significance is defined as a P value of ≤ 0.05. <0.05*, <0.01**, <0.001***, 0.0001****. **(A)** lactoferrin. **(B)** complement C3.

To determine whether the mutation of ERα calcium/metal interaction sites affects constitutive activity of D538G and cadmium induced expression of estrogen responsive genes, the calcium/metal interaction sites in D538G were mutated. HEK293T cells were transfected with the constitutively active mutant D538G or the calcium/metal interaction site mutants D538G/E542A/D545A, D538G/N532A, D538G/H516A/N519A/E523A, and D538G/C381A and treated with estradiol or cadmium and the amount of lactoferrin and complement C3 was measured. D538G was also treated with ICI-182,780. In the case of lactoferrin expression, cells transfected with D538G exhibited a 6.23-fold increase in lactoferrin mRNA when compared to wtERα confirming the constitutive activity of D538G (**Figure 6A**). Compared to cadmium treatment of wtERα, cadmium treatment of D538G resulted in an additional 5.06-fold increase in lactoferrin mRNA that was blocked by ICI-182,780 (**Supplementary 8A**). Mutation of the calcium/metal interaction sites resulted in an approximately 82%, 34%, 51%, and 67% decrease in constitutive activity of D538G/E542A/D545A, D538G/N532A, D538G/H516A/N519A/E523A, and D538G/C381A, respectively. Cadmium activation of D538G/E542A/D545A, D538G/N532A, D538G/H516A/N519A/E523A, and D538G/C381A was significantly lowered by approximately 85%, 40%, 62%, and 72%, respectively, compared to cadmium activation of D538G. In the case of compliment C3, cells transfected with D538G also exhibited a 2.33-fold higher expression of complement C3 mRNA compared to cells transfected with wtERα demonstrating the constitutive activity of D538G (**Figure 6B**). Treatment of D538G with cadmium resulted in an additional 1.68-fold increase in the expression of complement C3 mRNA compared to cadmium treatment of wtERα. Mutation of the calcium/metal interaction sites D538G/E542A/D545A, D538G/N532A, D538G/H516A/N519A/E523A, and D538G/C381A also resulted in a significant decrease in constitutive activity by approximately 73%, 44%, 49%, and 67%, respectively. Cadmium activation of D538G/E542A/D545A, D538G/N532A, D538G/H516A/N519A/E523A, and D538G/C381A was also significantly lower by approximately 77%, 28%, 56%, and 65%, respectively.

### Effects of cadmium on the recruitment of D538G and D538G calcium/metal interaction site mutants to the enhancers of estrogen responsive genes

**Figure 7:**
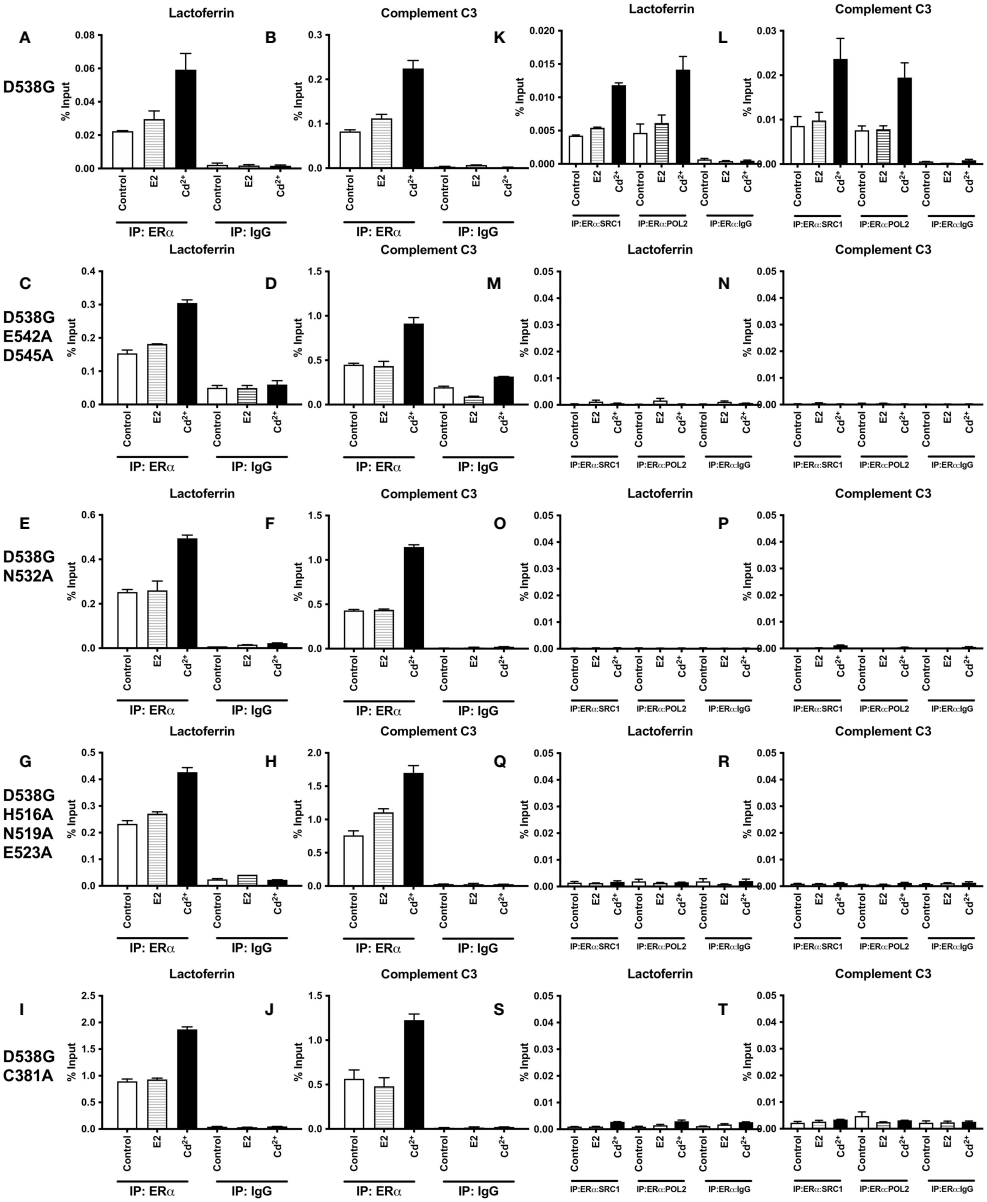
Effects of cadmium on the recruitment of D538G and D538G calcium/metal interaction site mutants to the enhancers of estrogen responsive genes. HEK293T cells were transfected with D538G ERα and mutants D538G/E542A/D545A, D538G/N532A, D538G/H516A/N519A/E523A, and D538G/C381A. Following transfection, the cells were treated with estradiol (100 nM) or cadmium (2 µM) for 60 minutes, crosslinked, lysed, and the occupancy of ERα on the enhancers of target genes was examined by ChIP assay. A Re-ChIP assay was performed, and the chromatin was first immunoprecipitated with an antibody to ERα, followed by immunoprecipitation with an antibody to SRC1, PolII, or IgG. DNA was quantified by real time qPCR and the recruitment of ERα, SRC1, and PolII is presented as percent input. The data are the mean of two independent experiments done in duplicate (mean ± SEM). **(A, B)** ChIP D538G. **(C, D)** ChIP D538G/E542A/D545A. **(E, F)** ChIP D538G/N532A. **(G, H)** ChIP D538G/H516A/N519A/E523A. **(I, J)** ChIP D538G/C381A. **(K, L)** Re-ChIP D538G. **(M, N)** Re-ChIP D538G/E542A/D545A. **(O, P)** Re-ChIP D538G/N532A. **(Q, R)** Re-ChIP D538G/H516A/N519A/E523A. **(S, T)** Re-ChIP D538G/C381A.

To determine whether cadmium treatment affects the recruitment of D538G and D538G calcium/metal interaction site mutants to the enhancers of estrogen responsive genes, cells were transfected with D538G, D538G/E542A/D545A, D538G/N532A, D538G/H516A/N519A/E523A, and D538G/C381A and treated for 60 minutes with estradiol or cadmium. ERα-DNA complexes were isolated and the recruitment of the mutants to the enhancers of lactoferrin and complement C3 was quantified. Compared to control, treatment of D538G, D538G/E542A/D545A, D538G/N532A, D538G/H516A/N519A/E523A, and D538G/C381A with estradiol did not increase the enrichment of the mutant receptors on the enhancers of lactoferrin (~1.34-fold) and complement C3 (~1.36-fold). However, treatment of D538G, D538G/E542A/D545, D538G/N532A, D538G/H516A/N519A/E523A, and D538G/C381A with cadmium resulted in an approximately 1.96- to 2.67-fold increase in the enrichment of receptors on the enhancers of lactoferrin and complement C3 (**Figures 7A–J**).

To determine whether a transcription complex is formed, a sequential ChIP assay was performed to determine the enrichment of SRC1 and PolII on the enhancers of lactoferrin and complement C3. Cells were again transiently transfected with D538G or the D538G calcium/metal interaction site mutants and treated for 60 minutes with estradiol or cadmium. Immunoprecipitation was first performed with an antibody to ERα and then with an antibody to either SRC1 or PolII and the enrichment of SRC1 and PolII on the enhancers of lactoferrin and complement C3 was quantified (**Figures 7K–T**). Treatment of D538G with estradiol did not further increase the enrichment of SRC1 and PolII on the enhancer of lactoferrin (~1.27- & 1.32-fold, respectively) or complement C3 (~1.15- & 1.02-fold, respectively). In contrast to estradiol, treatment with cadmium resulted in approximately 2.79- and 3.03-fold increase in enrichment of SRC1 and PolII on the enhancer of lactoferrin and an approximately 2.75- and 2.55-fold increase in enrichment on the enhancer of complement C3, respectively. However, treatment with estradiol or cadmium did not increase the enrichment of SRC1 or PolII on the enhancers of lactoferrin and complement C3 of ERα calcium/metal interaction site mutants D538G/E542A/D545A, D538G/N532A, D538G/H516A/N519A/E523A, and D538G/C381A.

### Effects of calcium, cadmium, calcium channel blockers, and calcium chelators on cell growth of MCF7 Y537S and MCF7 D538G cells

**Figure 8:**
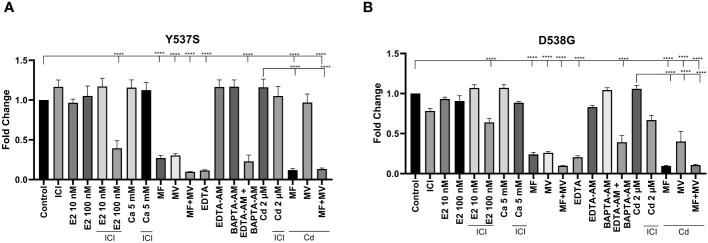
Effects of calcium, cadmium, ICI, calcium channel blockers and calcium chelators on cell growth. MCF7 Y537S and MCF7 D538G were treated with calcium (5 mM), cadmium (2 µM), ICI (1 µM), mibefradil (MF; 5 µM), methoxyverapamil (MV; 75 µM), EDTA (1 µM), BAPTA-AM (10 µM), EDTA-AM (1 µM) and E2 (10 nM or 100 nM) for 5 days, stained by crystal violet and counted by a plate reader. Using the 2^^-ΔΔCt^ method, data are presented as fold change compared to control, mean ± SEM; n=3. Statistical significance is defined as a P value of ≤ 0.05. 0.0001****. **(A)** MCF7 Y537S. **(B)** MCF7 D538G.

To ask whether calcium and/or cadmium contribute to the constitutive activity of Y537S and D538G, MCF-7 cells harboring Y537S and D538G ESR1 mutations (MCF7 Y537S and MCF7 D538G) were treated with calcium channel blockers or metal chelators. As expected, MCF-7 cells that express the constitutively active mutants grow in the absence and presence of estradiol (10 nM or 100 nM), calcium (5 mM), cadmium (2 µM), and the antiestrogen ICI-182-780 (1 µM) **Figure 8**). Treatment of MCF7 Y537S and MCF7 D538G cells with the calcium channel blockers mibefradil (MF; 5 µM) and methoxyverapamil (MV; 75 µM) and the extracellular calcium chelator EDTA (1 μM) resulted in an approximately 70-90% decrease in cell growth in MCF7 Y537S cells, approximately 73-90% decrease in cell growth in MCF7 D538G cells (**Figures 8A, B**). Treatment with the intracellular calcium chelators EDTA-AM (1 µM) and BAPTA-AM (10 µM) alone did not significantly reduce cell growth, but the combination treatment of EDTA-AM (1 µM) and BAPTA-AM (10 µM) significantly reduced cell growth in both MCF7 Y537S and MCF7 D538G cells by approximately 61-77%. Treatment of MCF7 Y537S and MCF7 D538G with cadmium had no significant effect on cell growth, but treatment with the channel blockers mibefradil and methoxyverapamil resulted in an approximately 70-73% decrease in the growth of MCF7 Y537S and approximately 74-76% decrease in the growth of MCF7 D538G consistent with the influx of cadmium through calcium channels. The ability of calcium channel blockers and chelators to decrease the growth of MCF7 cells expressing constitutively active mutants suggests that calcium and cadmium contribute to the constitutive activity of D538G and Y537S ESR1 mutants.

## Discussion

This study shows that cadmium activates ERα through a mechanism similar to calcium and contributes to and further increases the constitutive activity of ERα mutants Y537S and D538G. Mutational analysis identified four calcium/metal interaction sites: C381A, N532A, H516A/N519A/E523A, and E542/D545A in the LBD. Mutation of calcium/metal interaction sites in wtERa blocked the ability of cadmium to activate the receptor. Mutation of calcium/metal interaction sites in the mutants Y537S and D538G led to a significant decrease in constitutive activity and cadmium induced activation. Mutation of the calcium/metal interaction sites in Y537S and D538G did not inhibit DNA binding but prevented a stable interaction with SRC1 and PolII. Additionally, growth assays indicated that treatment with calcium channel blockers and chelators significantly reduced the growth of MCF7 cells harboring these constitutively active mutations, highlighting the critical role of calcium in the growth regulation of these cells.

Cadmium is a metal with no known physiological function that activates ERα. Exposure to the metal occurs primarily through dietary sources, cigarette smoking, and, to a lesser degree, drinking water (26). Cadmium has a long biological half-life, estimated to be between 4-38 years (27). The ability to activate ERα and long biological half-life suggests that exposure to cadmium not only increases the risk of developing breast cancer (6, 28, 29) but affects the response to endocrine therapy.

In the classical genomic pathway, ERα is activated by the binding of estradiol within the pocket of the LBD. As a result, ERα dissociates the heat shock complex, localizes in the nucleus, dimerizes and binds to DNA, and recruits coactivators and the general transcriptional machinery. ERα consists of the N-terminal domain that contains activation function-1 (AF-1), the central DNA binding domain, hinge region, and the C-terminal domain that contains the LBD and AF-2. The LBD includes 11 α-helices (H1, H3-H12) (30–32). Based on the crystal structure of the retinoic acid receptor in the absence and presence of its ligand, it is thought that several major conformational changes occur in the LBD of nuclear receptors as a result of ligand binding (33). In the absence of ligand, a short loop separates helices H10 and H11 while helix H12 flanks the binding pocket. Following the binding of ligand, helix H3 rotates around its axis and bends toward the pocket and helix H4. This rearrangement rotates helix H11 around its axis to form the contiguous helix H10/H11 creating an extended loop between helices H11 and H12 and rotating helix H12 over the pocket. Helix H10/H11 together with helices H8 and H9 creates the dimerization domain and helices H3 and H12 together with helix H4 creates the AF-2 domain (31, 34).

**Figure 9:**
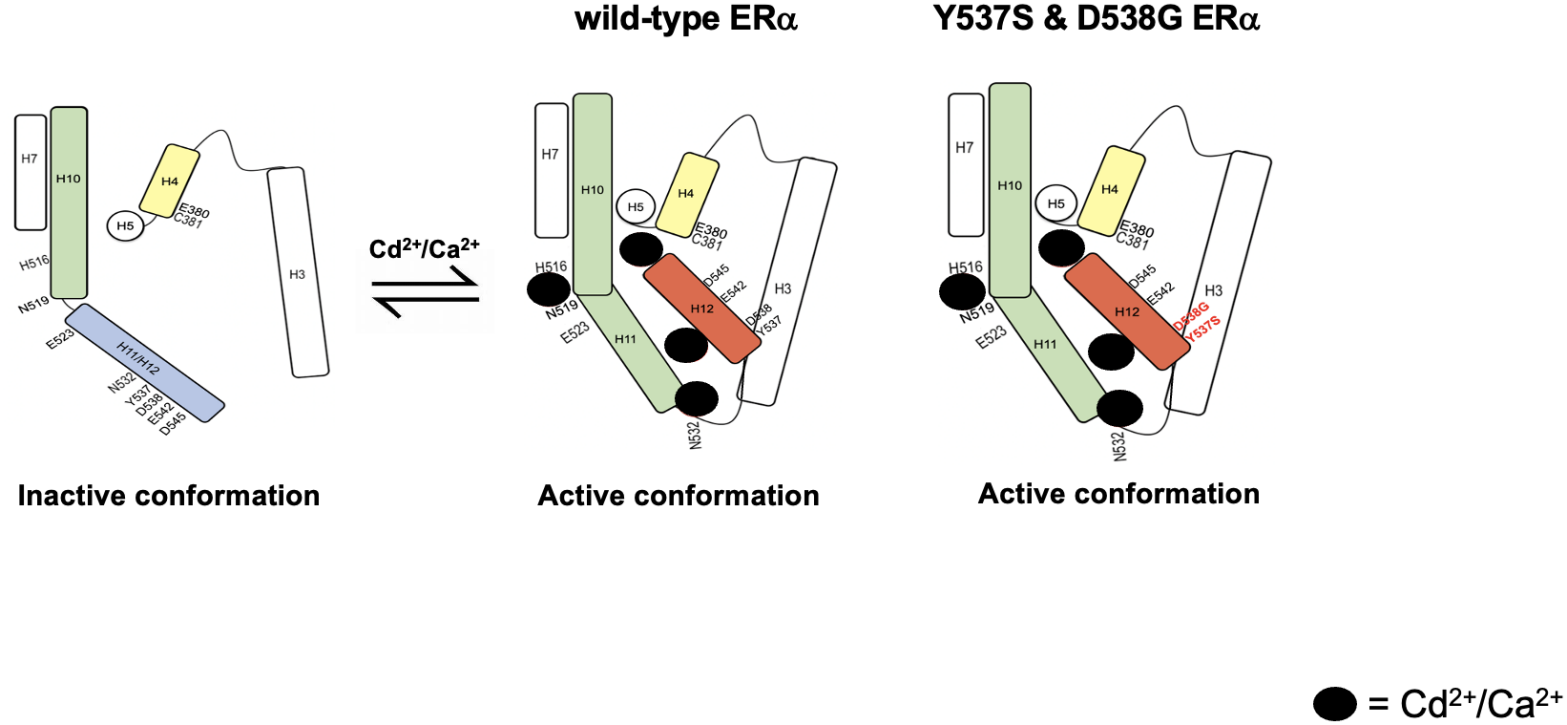
Schematic summarizing the proposed mechanism of ERα activation and the activation of constitutively active ERα mutants Y537S and D538G.

In contrast to estradiol binding in the pocket, calcium activates ERα through four interaction sites on the solvent accessible surface of the LBD (4), (3). Mutational analysis and molecular modeling suggested that the first site is formed by a direct interaction of calcium with the side chains of H516 on helix H10, N519 in the loop between helices H10 and H11, and E523 on helix H11; the second site is formed by a direct interaction of calcium with the backbone of K529 on helix H11 and the side chain of N532 and backbone of V534 in loop 11-12; the third site is formed by a direct interaction of calcium with the side chains of D538, E542, and D545 on helix H12; and the fourth site is formed by the direct interaction of calcium with the side chains of H377, E380, and C381 on helix H4. Cadmium is a bivalent cation with an ionic radius similar to calcium. The results of the present study further define the cadmium interaction sites and shows that the calcium interaction sites are the cadmium interaction sites, which are important for the activation of ERα and the constitutive activity of Y537S and D538G. The identification of calcium/metal interaction sites at the ends of helices H10, H11, and H12 and on H4 suggests a model whereby calcium and cadmium alter and/or stabilize the α-helices in the LBD to induce conformation changes similar to the conformational changes proposed for estradiol (35). In the proposed model (**Figure 9**), the interaction of cadmium and calcium with the side chains of amino acids at the C-terminal end of helix H10, in the loop between helices H10 and H11, and on the N-terminal end of helix H11 (H516, N519, and E523) causes helix H11 to rotate around its axis to form a continuous helical structure with helix H10 contributing to the formation of the dimerization domain. The interaction of the metals with the backbone and side chains of amino acids on the C-terminal end of helix H11 and in the loop between helices H11 and H12 (N532) as well as the interaction at the N-terminal end of helix H12 (E542 and D545) repositions helix H12 over the binding pocket while the interaction on helix H4 (C381) induces a kink in the helix formed by helices H4 and H5 facilitating the movement of helix H3 towards helix H4 and the ligand binding pocket to form the coactivator binding site (31, 34).

Other studies suggest that a spring force originating from the strain in the loop between helix H11-H12 of wtERα opposes the folding of H12 into the active conformation and keeps the pocket open for ligand binding (Mayne et al.). The spring force is abrogated by a latching mechanism in the Y537S mutant and by relaxing the folded form of the loop in the D538G mutant thereby enabling formation of the active conformation. In fact, Y537S has been shown to form a hydrogen bond with D351 (21) facilitating a tighter packing of the H11-H12 loop against the LBD while D538G relaxes the helical character of the N-terminal end of helix H12, thus relaxing the H11-12 loop and improving the packing of its hydrophobic side chains (22). Clustering of mutations within the loop connecting helix H11 and H12 suggests that the loop also provides an energetic barrier that controls the conformational switch of helix H12 that regulates coactivator binding (Mayne et al.). Collectively, these studies suggest that the constitutive activity of Y537S and D538G stems from their ability to adopt a stable agonist conformation in the absence of hormone (36). We propose a model where cadmium also contributes to and enhances the constitutive activity of Y537S and D538G. In support of the role of cadmium in the constitutive activity of Y537S and D538G, loss of the calcium/metal interaction sites results in diminished constitutive activity. The constitutively active mutants bind to DNA and recruit SRC1 and PolII, however, in the absence of the calcium/metal sites, the Y537S and D538G mutants bind to DNA but do not recruit SRC1 or PolII, which may be due to the lack of an appropriate conformation of the LBD. Although decreased affinity and/or stability of the receptor/coregulator complex cannot be ruled out, the results show that intact calcium/metal interaction sites are important for the constitutive activity of Y537S and D538G. In support of the ability of cadmium to increase the transcriptional activity of Y537S and D538G through sites on the aqueous surface of the LBD, estradiol which binds in the hydrophobic pocket did not increase the transcriptional activity of the constitutively active mutants, whereas cadmium which binds on the surface increased the transcriptional activity of the mutants. This is consistent with our mutational analysis, molecular modeling, and binding assays showing that metals interact with sites on the surface of the LBD and noncompetitively block estradiol binding to the receptor (11). The current model also proposes that the four calcium/metal interaction sites are important for inducing an active conformation of the LBD and predicts that the site between helix H10, loop H10-H11, and helix H11 (H516/N519/E523) is involved in the rotation of helix H11 and the formation of the dimerization domain. In support of a role of H516/N519/E523 in the formation of the dimerization domain, cadmium failed to activate Y537S/H516A/N519A/E523A, whereas estradiol activated the mutant suggesting that the binding of estradiol in the hydrophobic pocket rotates helix H11 and compensates for the loss of the metal interaction site on the surface of the LBD. Additionally, the loss of the calcium/metal interaction sites at the C-terminal end of helix H11 and loop between helices H11 and H12 (N532A) and at the N-terminal end of helix H12 (E542A/D545A) prevents the repositioning of helix H12 over the LBD pocket by abrogating the spring force that originates from the strain of the H11-H12 loop similar to what is proposed for the constitutively active mutants (37). The mutation of C381 on helix H4 prevents the formation of a kink in the helix formed by helices H4 and H5 and the formation of the coactivator binding site. Unlike H516A/N519A/E523A, estradiol does not compensate for the loss of the metal interaction sites at N532A, E542A/D545A, and C381A in the constitutively active mutants.

In summary, our results demonstrate that cadmium activates ERα through calcium/metal interaction sites. Mutation of calcium/metal interaction sites in the ERα mutants Y537S and D538G results in a decrease of constitutive activity suggesting that the intracellular environment, presumably intracellular calcium and cadmium, contributes to the constitutive activity of these mutants. As cadmium activates ERα through the solvent accessible surface of the LBD, it enhances the constitutive activity of Y537S and D538G whereas estradiol does not. Taken together, the results suggest that exposure to cadmium plays a role in the etiology and response to treatment of breast cancer due, in part, to its ability to activate ERα. As cadmium mimics calcium, the results support further investigation into the inclusion of calcium channel blockers with existing therapies to treat ER positive metastatic breast cancers.

## Data availability statement

The datasets presented in this study can be found in online repositories. The names of the repository/repositories and accession number(s) can be found in the article/**Supplementary Material**.

## Author contributions

JP: Conceptualization, Data curation, Formal analysis, Investigation, Methodology, Project administration, Visualization, Writing – original draft, Writing – review & editing. QW: Data curation, Methodology, Validation, Visualization, Writing – review & editing. GY: Data curation, Methodology, Writing – review & editing. RG: Methodology, Writing – review & editing. NH: Data curation, Methodology, Writing – review & editing. BH: Conceptualization, Funding acquisition, Writing – review & editing. MM: Conceptualization, Data curation, Formal analysis, Funding acquisition, Investigation, Methodology, Project administration, Resources, Supervision, Validation, Visualization, Writing – review & editing.
