# Supplementary figures and images for: Cadmium activation of wild-type and constitutively active estrogen receptor alpha
Source: Front Endocrinol (Lausanne). 2024 Aug 9;15:1380047. doi: 10.3389/fendo.2024.1380047 (PMC11341946; doi:10.3389/fendo.2024.1380047)

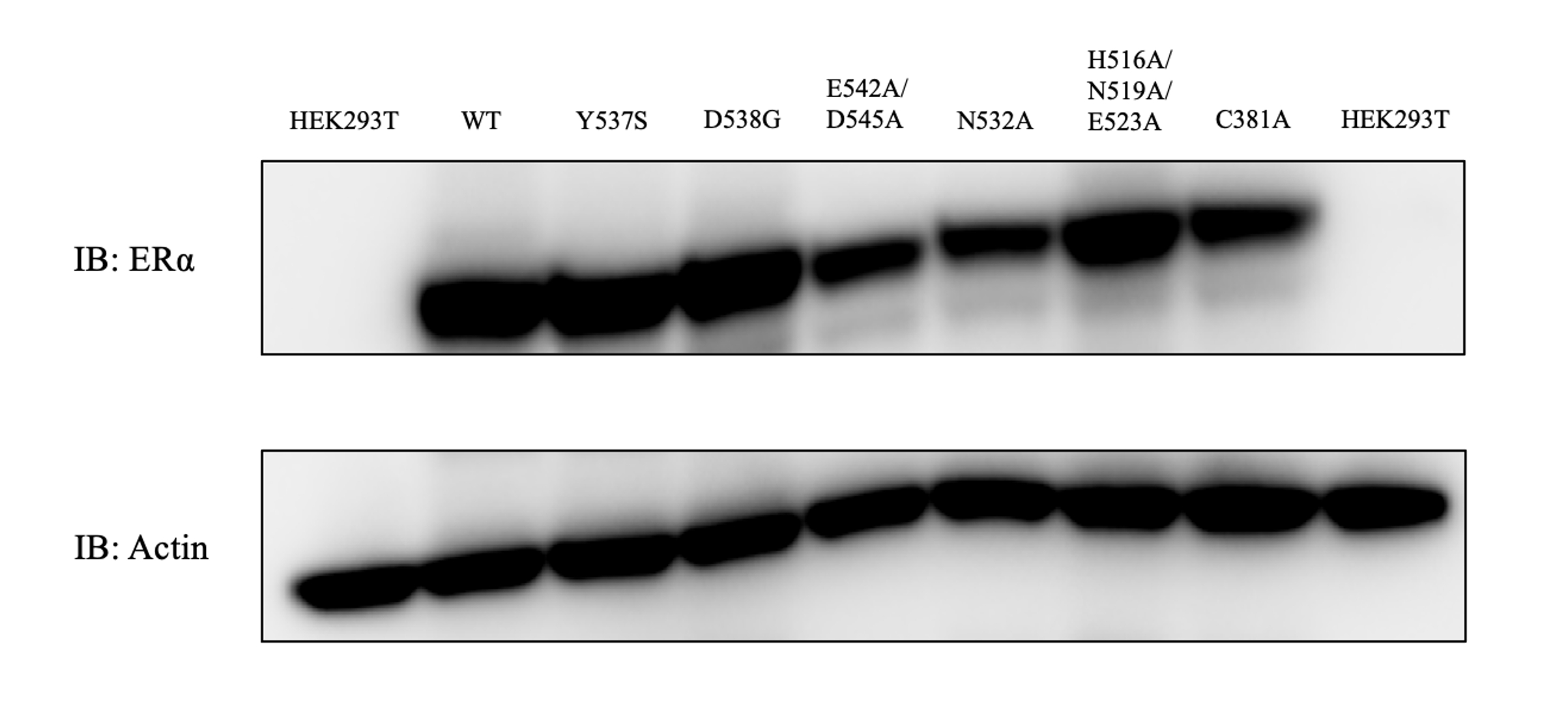

Supplement: Supplementary file 1 [file Image_1.tiff]

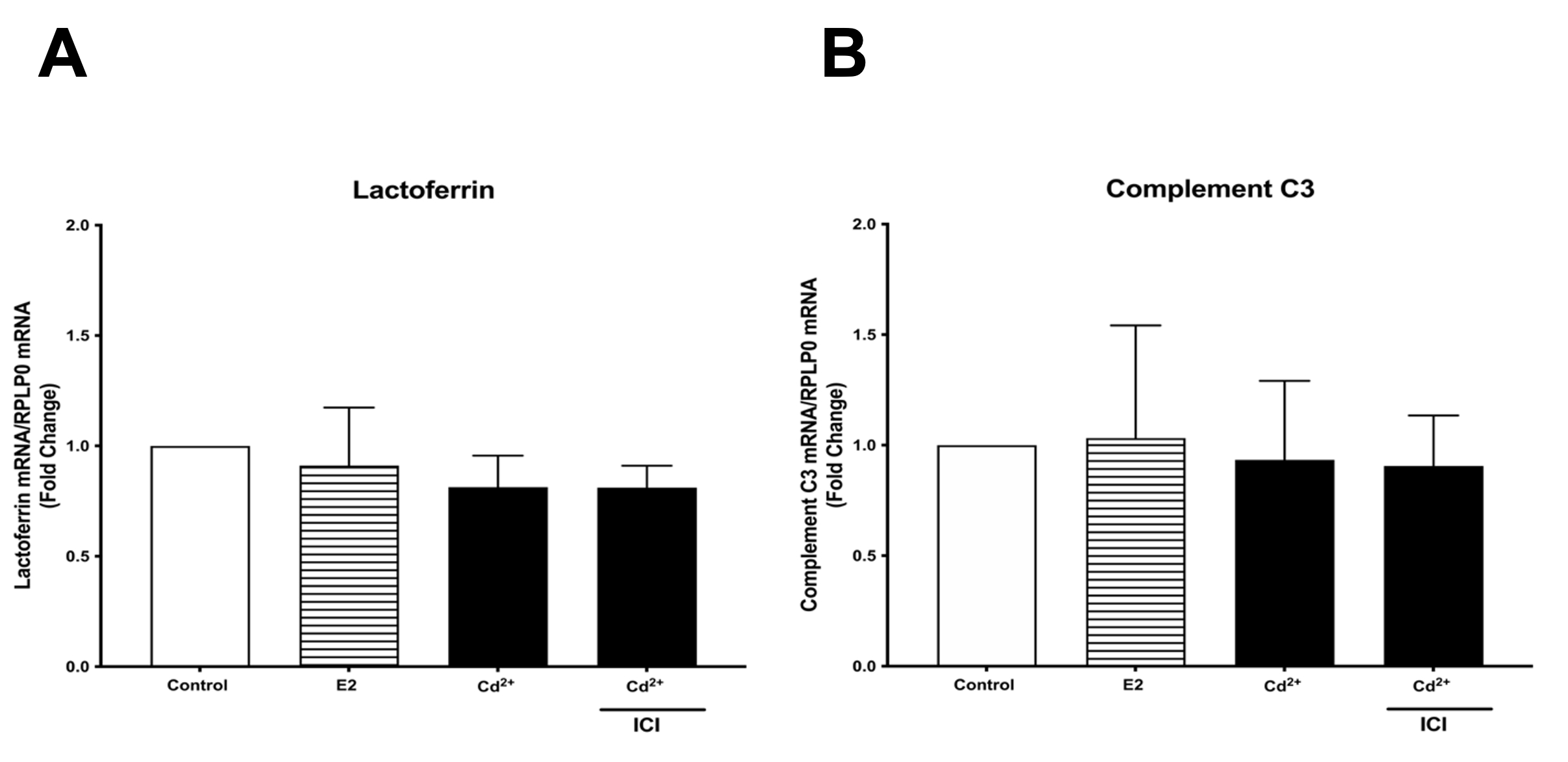

Supplement: Supplementary file 2 [file Image_2.tiff]

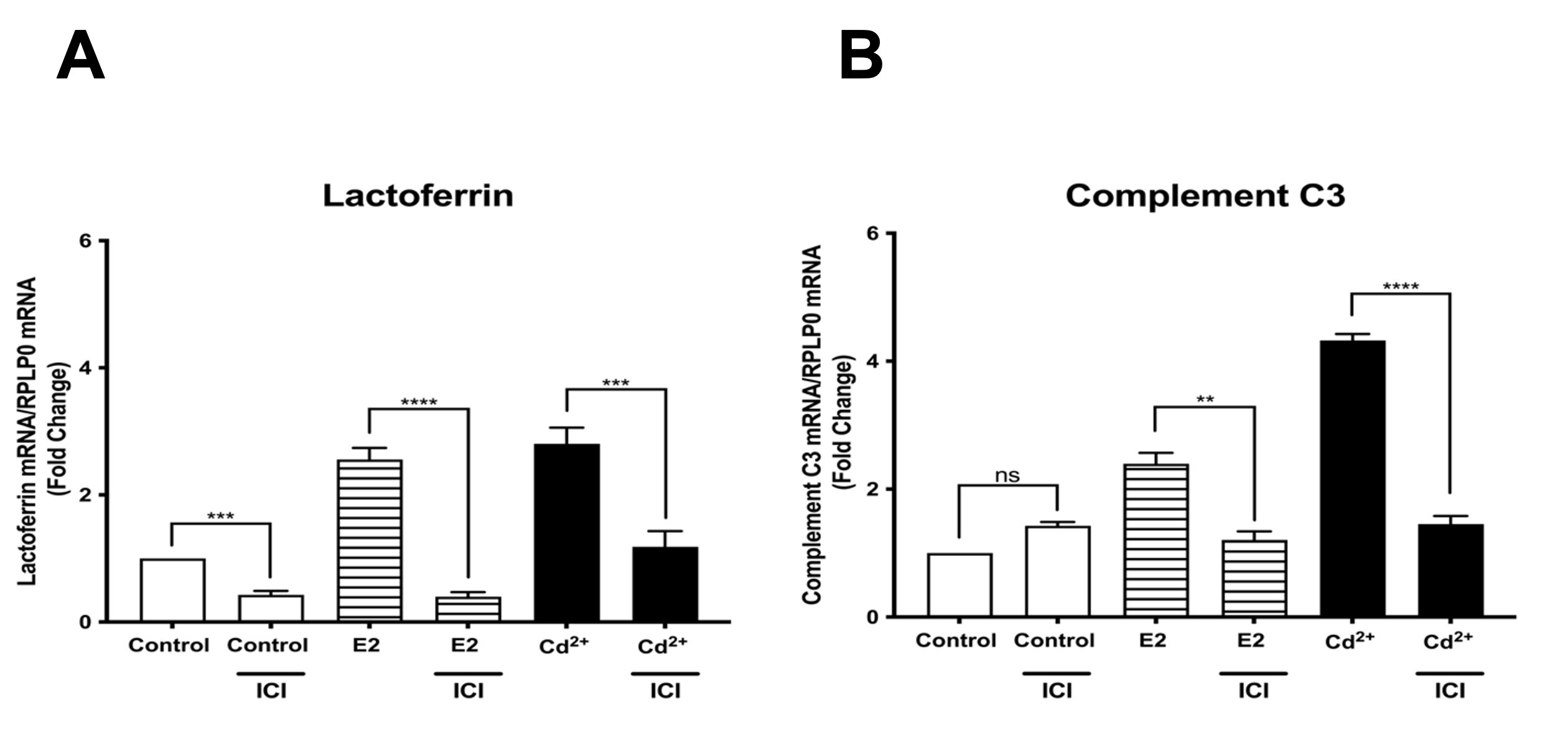

Supplement: Supplementary file 3 [file Image_3.tiff]

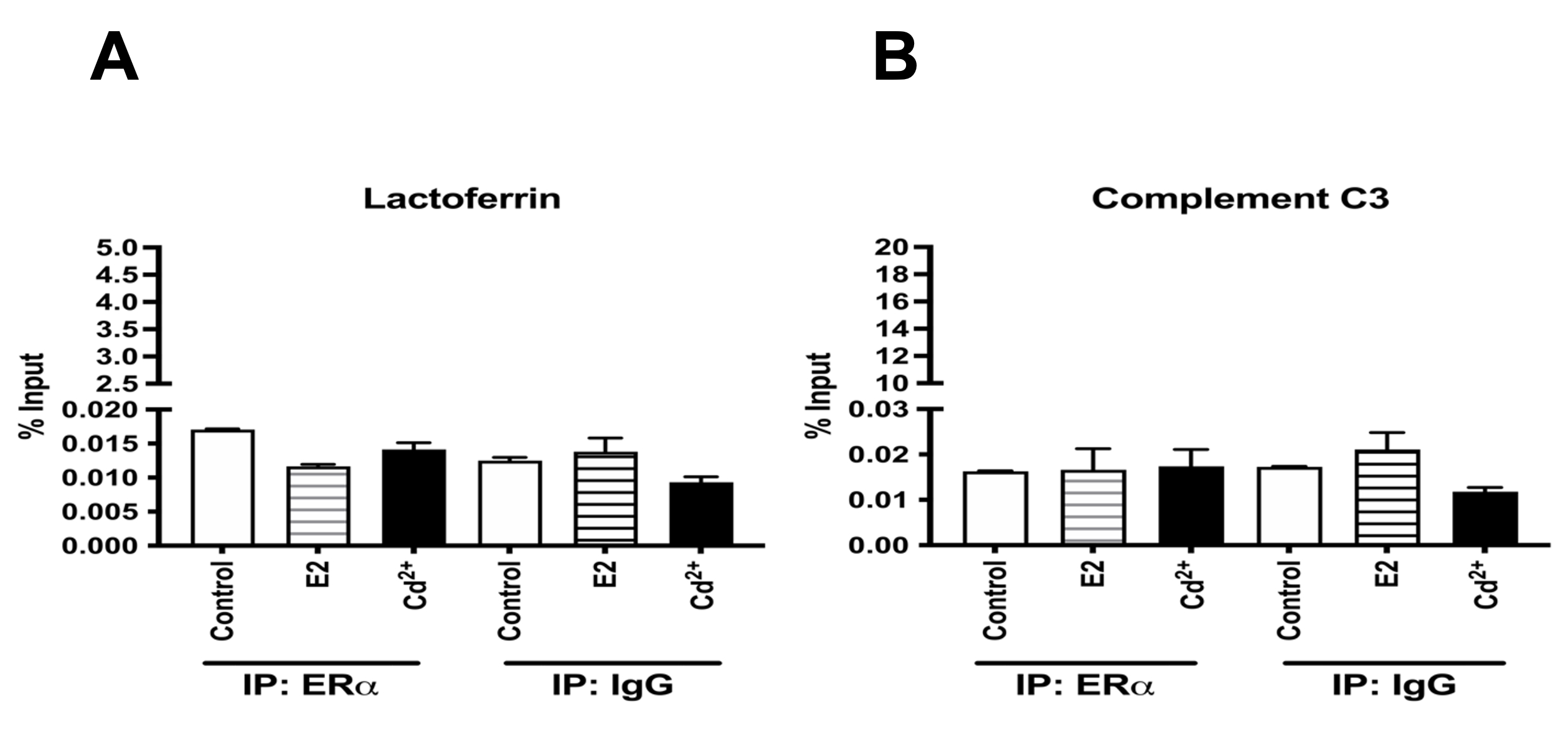

Supplement: Supplementary file 4 [file Image_4.tiff]

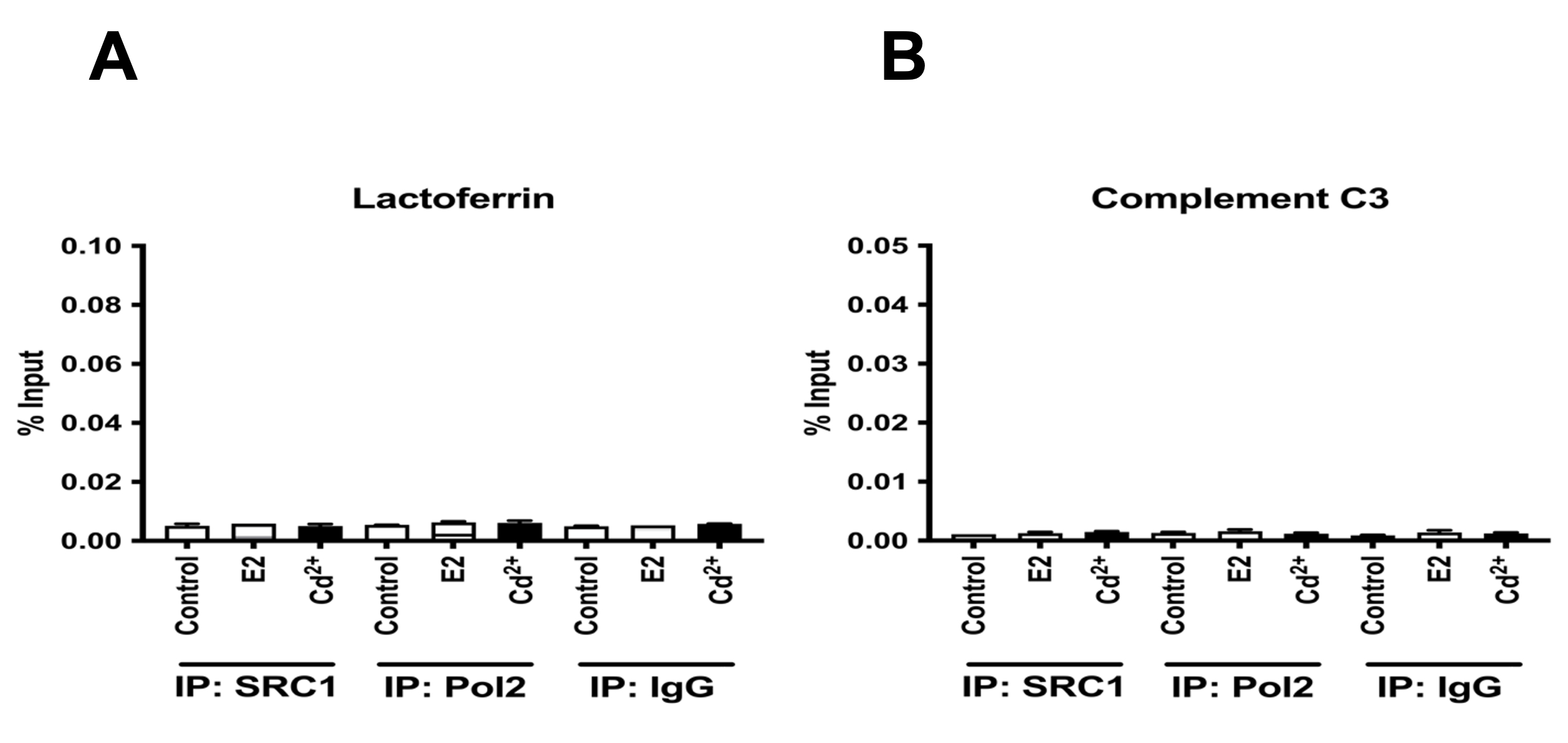

Supplement: Supplementary file 5 [file Image_5.tiff]

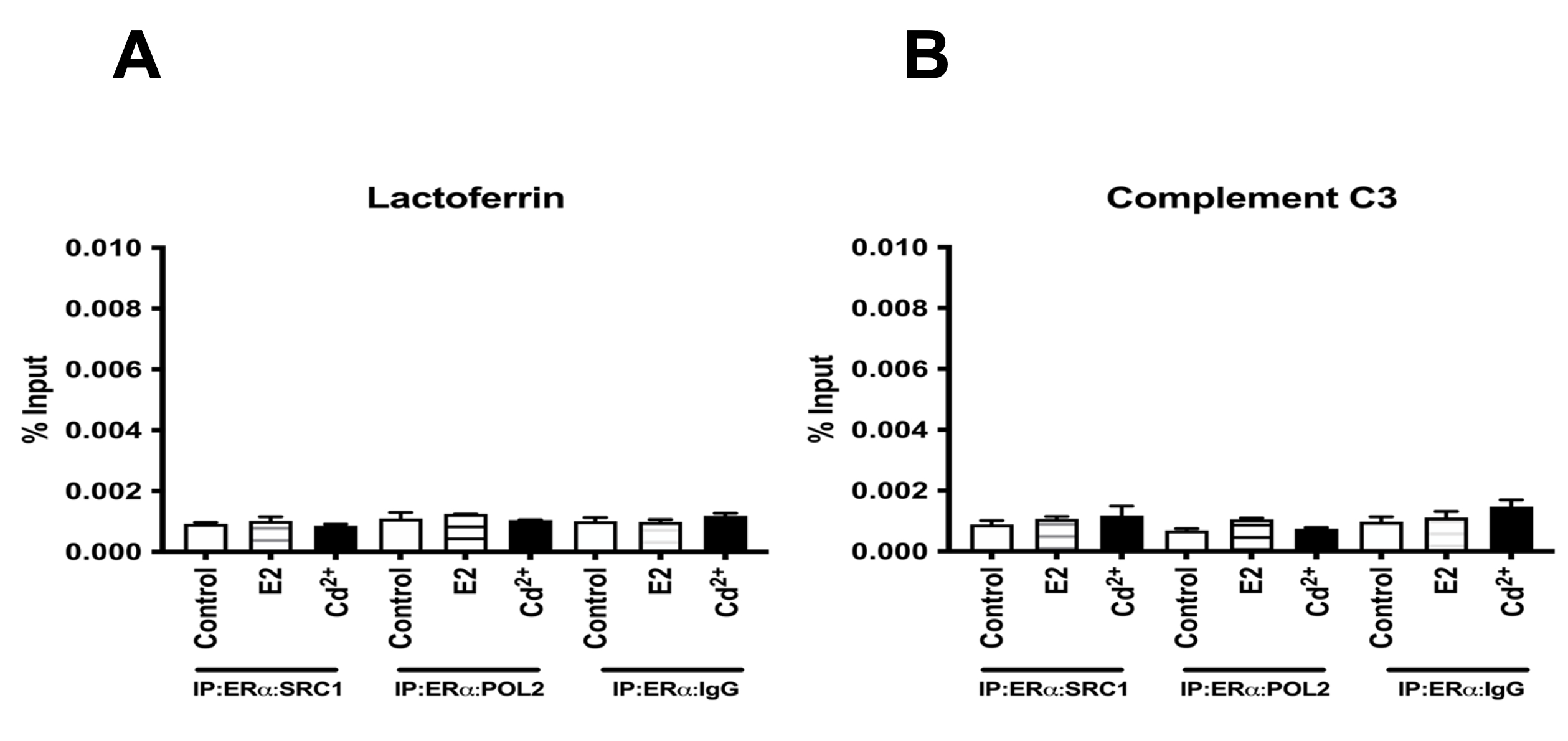

Supplement: Supplementary file 6 [file Image_6.tiff]

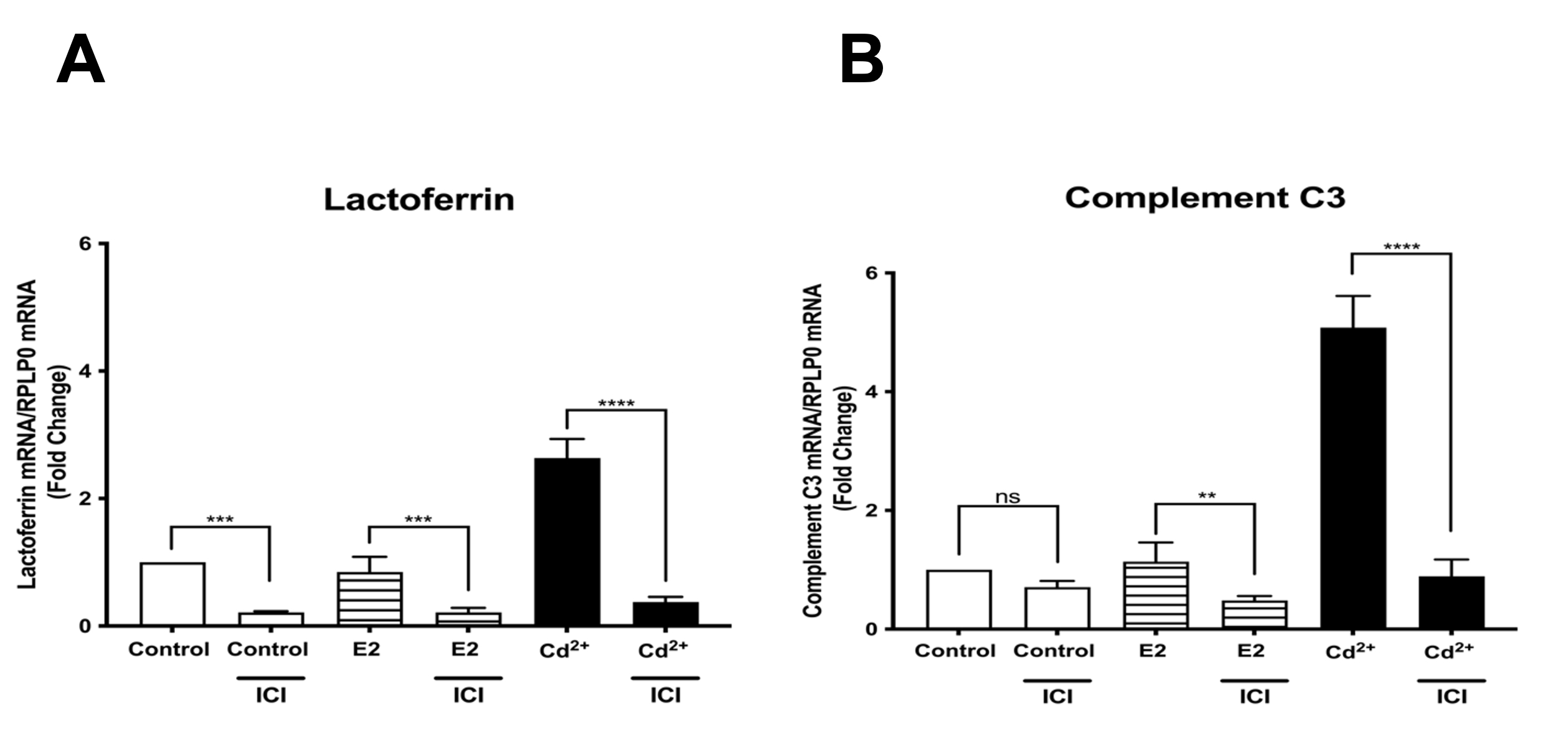

Supplement: Supplementary file 7 [file Image_7.tiff]

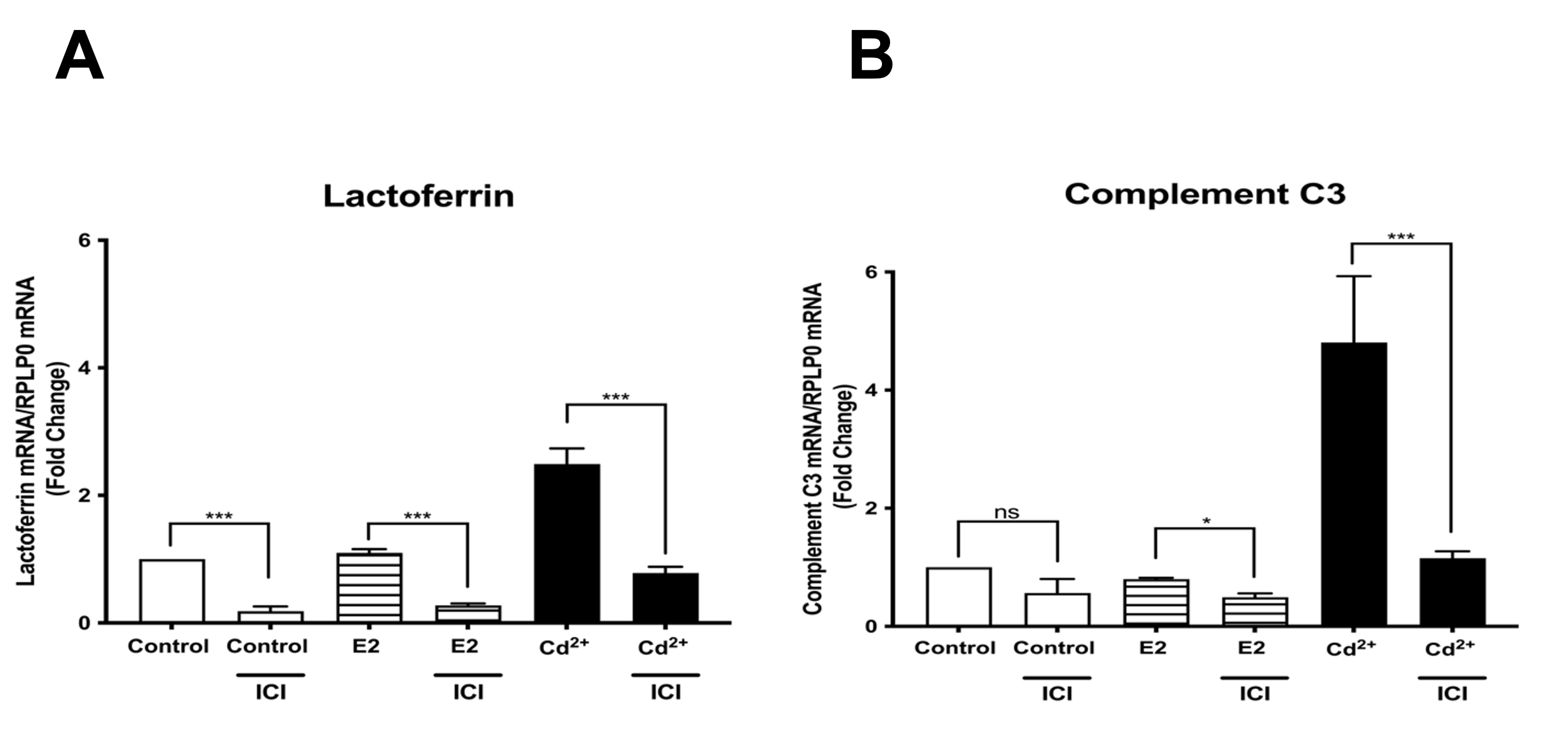

Supplement: Supplementary file 8 [file Image_8.tiff]
