# Supplementary material for: Cadmium activation of wild-type and constitutively active estrogen receptor alpha
Source: Front Endocrinol (Lausanne). 2024 Aug 9;15:1380047. doi: 10.3389/fendo.2024.1380047 (PMC11341946; doi:10.3389/fendo.2024.1380047)
Supplement: Supplementary file 9 [file DataSheet_1.docx]

**Supplement Figure Legends**

**Supplement 1.** Expression of exogenous wild-type and mutant ERα in HEK293T cells. HEK293T cells were transiently transfected with wild-type ERα and ERα mutants Y537S, D538G, E542A/D545A, N532A, H516A/N519A/E523A, and C381A. Forty-eight hours post-transfection, the cells were lysed and centrifuged. The lysate was run on a 10% SDS-PAGE gel, transferred to a nitrocellulose membrane, and incubated with anti-estrogen receptor-alpha antibody. The target protein signals were then visualized on an Amersham Imager 600. Representative blot from three independent experiments.

**Supplement 2.** Effects of cadmium on ERα target genes in untransfected HEK293T cells. HEK293T cells were treated with estradiol (100 nM) or cadmium (2 µM) in the absence or presence of ICI-182,780 (fulvestrant; 1 µM) for 48 hours. The RNA was isolated, the amount of lactoferrin and complement C3 mRNA was measured by real time qPCR and normalized to the amount of ribosomal protein P0 (RPLP0) mRNA. Using the 2^^-ΔΔCt^ method, data are presented as fold change compared to control, mean ± SEM; n=3. Statistical significance is defined as a P value of ≤ 0.05. *p≤0.05; **p≤0.01; ***p≤0.001; ****p≤0.0001. (**A**) lactoferrin. (**B**) complement C3.

**Supplement 3.** Effects of cadmium on ERα target genes in transfected cells. HEK293T cells were transiently transfected with wild-type ERα. Following transfection, the cells were treated with estradiol (100 nM) or cadmium (2 µM) in the absence or presence of ICI-182,780 (fulvestrant; 1 µM) for 48 hours. The RNA was isolated, the amount of (**A**) lactoferrin and (**B**) complement C3 mRNA was measured by real time qPCR and normalized to the amount of ribosomal protein P0 (RPLP0) mRNA. Using the 2^^-ΔΔCt^ method, data are presented as fold change compared to control, mean ± SEM; n=3. Statistical significance is defined as a P value of ≤ 0.05. *p≤0.05; **p≤0.01; ***p≤0.001; ****p≤0.0001. (**A**) lactoferrin. (**B**) complement C3.

**Supplement 4.** Effects of cadmium on the recruitment of ERα to the enhancers of target genes in untransfected HEK293T cells. HEK293T cells were treated with estradiol (100 nM) or cadmium (2 µM) for 60 minutes, crosslinked, lysed, and the occupancy of ERα on the enhancers of target genes was examined by ChIP assay. DNA was quantified by real time qPCR and the recruitment of ERα is presented as percent input. The data are the mean of duplicate experiments. (**A**) lactoferrin. (**B**) complement C3.

**Supplement 5.** Effects of cadmium on the recruitment of SRC1 and PolII to the promoters of ERα target genes in untransfected HEK293T cells. HEK293T cells were treated with estradiol (100 nM) or cadmium (2 µM) for 60 minutes, crosslinked, and lysed, and the occupancy of SRC1 and PolII on the promoter of ERα target genes was examined by ChIP assay. The chromatin was immunoprecipitated with an antibody to SRC1, PolII, or IgG. DNA was quantified by real time qPCR, and the recruitment of proteins of SRC1 and PolII is presented as percent input. The data are the mean of duplicate experiments. (**A**) lactoferrin. (**B**) complement C3.

**Supplement 6.** Effects of cadmium on the recruitment of SRC1 and PolII to the promoters of ERα target genes in untransfected HEK293T cells. HEK293T cells were treated with estradiol (100 nM) or cadmium (2 µM) for 60 minutes, crosslinked, and lysed. The chromatin was first immunoprecipitated with an antibody to ERα, followed by immunoprecipitation with an antibody to SRC1, PolII, or IgG. DNA was quantified by real time qPCR, and the recruitment of SRC1 and PolII is presented as percent input. The data are the mean of duplicate experiments. (**A**) lactoferrin. (**B**) complement C3.

**Supplement 7.** Effects of cadmium on ERα target genes in Y537S transfected cells. HEK293T cells were transiently transfected with ERα Y537S. Following transfection, the cells were treated with estradiol (100 nM) or cadmium (2 µM) in the absence or presence of ICI-182,780 (fulvestrant; 1 µM) for 48 hours. The RNA was isolated, the amount of (**A**) lactoferrin and (**B**) complement C3 mRNA was measured by real time qPCR and normalized to the amount of ribosomal protein P0 (RPLP0) mRNA. Using the 2^^-ΔΔCt^ method, data are presented as fold change compared to control, mean ± SEM; n=3. Statistical significance is defined as a P value of ≤ 0.05. *p≤0.05; **p≤0.01; ***p≤0.001; ****p≤0.0001. (**A**) lactoferrin. (**B**) complement C3.

**Supplement 8.** Effects of cadmium on ERα target genes in D538G transfected cells. HEK293T cells were transiently transfected with ERα D538G. Following transfection, the cells were treated with estradiol (100 nM) or cadmium (2 µM) in the absence or presence of ICI-182,780 (fulvestrant; 1 µM) for 48 hours. The RNA was isolated, the amount of (**A**) lactoferrin and (**B**) complement C3 mRNA was measured by real time qPCR and normalized to the amount of ribosomal protein P0 (RPLP0) mRNA. Using the 2^^-ΔΔCt^ method, data are presented as fold change compared to control, mean ± SEM; n=3. Statistical significance is defined as a P value of ≤ 0.05. *p≤0.05; **p≤0.01; ***p≤0.001; ****p≤0.0001. (**A**) lactoferrin. (**B**) complement C3.
